# Application of Immunotherapy in Hepatocellular Carcinoma

**DOI:** 10.3389/fonc.2021.699060

**Published:** 2021-08-26

**Authors:** Lele Miao, Zhengchao Zhang, Zhijian Ren, Yumin Li

**Affiliations:** ^1^Department of General Surgery, Second Hospital of Lanzhou University, Lanzhou, China; ^2^Key Laboratory of the Digestive System Tumors of Gansu Province, Second Hospital of Lanzhou University, Lanzhou, China

**Keywords:** hepatocellular carcinoma, immunotherapy, immune checkpoint inhibitors, tumor vaccines, adoptive cell therapy

## Abstract

Hepatocellular carcinoma is one of the most common malignancies globally. It not only has a hidden onset but also progresses rapidly. Most HCC patients are already in the advanced stage of cancer when they are diagnosed, and have even lost the opportunity for surgical treatment. As an inflammation-related tumor, the immunosuppressive microenvironment of HCC can promote immune tolerance through a variety of mechanisms. Immunotherapy can activate tumor-specific immune responses, which brings a new hope for the treatment of HCC. At the present time, main immunotherapy strategies of HCC include immune checkpoint inhibitors, tumor vaccines, adoptive cell therapy, and so on. This article reviews the application and research progress of immune checkpoint inhibitors, tumor vaccines, and adoptive cell therapy in the treatment of HCC.

## Introduction

Primary liver cancer is one of the common malignant tumors, and its main pathological type is hepatocellular carcinoma (HCC). According to the 2018 cancer statistics of the World Health Organization, the incidence of liver cancer ranks 6th and the mortality rate ranks 4th among the most common cancers in the world (stomach cancer ranks third with a slight advantage) (1). The latest cancer statistics in 2020 show that the incidence of liver cancer still ranks sixth among the most common cancers in the world, but its mortality rate has risen from the fourth to the third (significantly exceeding the mortality rate of stomach cancer) (2) The traditional treatment mainly includes surgery, radiotherapy, chemotherapy, radiofrequency ablation (RFA), intervention, and targeted therapy. Multidisciplinary comprehensive treatment is an effective treatment strategy for prolonging the survival time of patients with HCC. However, the current 5-year survival rate of HCC patients after surgery is only about 36.9% (3), and the 5-year recurrence rate is as high as 70% (4). This is closely related to its tumor microenvironment. HCC is a typical inflammatory-related tumor. Its microenvironment contains a large number of macrophages, innate immune cells, and adaptive immune cells, forming a complex immune tolerance microenvironment (5, 6). Besides, the liver itself is a special immune-tolerant organ that can effectively escape the immune response (7). In recent years, immunotherapy has gradually become an important treatment for HCC. Tumor immunotherapy can enhance the immune response of the body, stimulate tumor-specific immunity, reactivate immune cells, and finally achieve the purpose of anti-tumor. Common tumor immunotherapy includes immune checkpoint inhibitors, tumor vaccines, and adoptive cell therapy. This paper reviews the application and research progress of immune checkpoint inhibitors, tumor vaccines, and adoptive cell therapy in the treatment of HCC. It aims to provide some references for clinicians in the treatment of HCC.

## Immunosuppressive Mechanisms and Immune Escape in HCC

The liver has a complex immune microenvironment. The liver is continuously exposed to various antigens passing through the portal vein, especially those from intestinal tract. Therefore, the liver microenvironment continues to show immune tolerance, which is to inhibit inappropriate inflammatory reaction and prevent autoimmune liver injury (8, 9). The specific immune system of HCC and tumor cells constitute a special immune tolerance microenvironment, which can protect tumor cells from the attack of their own immune system and promote the immune escape of tumor cells (10). The immunosuppressive mechanism of HCC is not completely clear at present, which may be related to the following mechanisms:

- The occurrence and progression of HCC are usually accompanied by chronic inflammation (e.g. viral hepatitis B and C) and chronic disease (e.g. liver cirrhosis). Under the action of long-term inflammation, many inhibitory cytokines (e.g. IL-10, IL-35 and TGF-β) are constantly produced, and a large number of immunosuppressive cells, such as regulatory T cells (Tregs), M2 macrophages, and myeloid-derived suppressor cells (MDSCs), are recruited into the liver (11). Furthermore, some immunosuppressive cells of the liver itself are activated or normal cells are transformed into immunosuppressive cells (10). These inhibitory cytokines and immunosuppressive cells together form the immunosuppressive microenvironment of HCC (11).
- Immunosuppressive cells in tumor tissue can promote HCC tolerance (12, 13). Tumor-associated monocytes, for example, can significantly increase the glycolysis level in the area around the tumor. Activation of glycolysis induced these cells to express PD-L1 (through NF-κB signaling pathway) and decreased the function of cytotoxic T lymphocyte (13).
- Tumor-associated macrophages (TAMs), as one of the key components constituting the immunosuppressive microenvironment of HCC, not only cannot eliminate tumor cells, instead will promote tumor growth (14, 15).
- HCC cells can release some cytokines, such as 14-3-3ζ, which can destroy the activation, proliferation and anti-tumor function of tumor-infiltrating T lymphocytes (TILs) (16). Beyond that, overexpression of 14-3-3ζ can also differentiate naive T cells from effector T cells to Tregs (16).
- The expression of immune checkpoints in HCC tissues is increased (5, 17, 18). The combination of immune checkpoints and their respective ligands will inhibit the activation and proliferation of T cells.
- Activation or alteration of some genes and signaling pathways may promote immune escape in HCC (19). For example, activation of β-Catenin (20) or mutation of *CTNNB1* (21) may promote immune escape in HCC.
- Epithelial-to-mesenchymal-transition (EMT) can induce the up-regulation of PD-L1, PD-L2, CD73 and B7-H3; and reversing EMT can inhibit the expression of these markers (22).

In response to these mechanisms, some corresponding measures can be taken to block, inhibit or reverse these mechanisms. For instance, measures can be taken to neutralize inhibitory cytokines or prevent their production. Yang et al. (23) found that reducing the level of IL-35 could reduce the metastasis of HCC and improve overall survival (OS) of HCC patients. HCC patients with high expression of PD-1/PD-L1 can be treated with corresponding immune checkpoint inhibitors (ICIs). Combination therapy based on PD-1/PD-L1 inhibitors can promote the response of antigen-specific CD8+ T cells in HCC (24). For TAMs, some special methods (for example, modulatory miRNA methods and immune checkpoint blockade) can be used to repolarize TAMs to the anti-HCC phenotypes (25). For the moment, these coping strategies mostly stay in theoretical and preclinical studies. With the rapid development of tumor immunotherapy, it has gradually become one of the important methods for the treatment of HCC in recent years. These immunotherapy mainly include ICIs, tumor vaccines and adoptive cell therapy, especially ICIs are used more frequently.pt?>

## Possible Resistance Mechanisms Related to the Immunotherapy of HCC

In recent years, unprecedented progress has been made in tumor immunotherapy. Drugs, therapies and strategies related to tumor immunotherapy are also emerging one after another. Nevertheless, the low response rate and the consequent resistance problem have greatly limited the efficacy of immunotherapy (26). These mechanisms of immunotherapy resistance can be divided into the intrinsic mechanisms and the extrinsic mechanisms. The intrinsic mechanisms include (27, 28): a. The activation of MAPK signaling pathway leads to the production of VEGF and IL-8 (inhibiting the recruitment and function of effector T cells) (29); b. Loss of PTEN expression leads to enhancement of PI3K signaling pathway, which is negatively correlated with gene expression of IFNγ and CD8+ T cell infiltration (30, 31); c. The continuous activation of WNT/β-catenin signaling pathway hinders the homing of T cells (32); d. Up-regulation of PD-L1 expression on the surface of tumor cells inhibits the anti-tumor effect of effector T cells; e. Decreased antigen presentation ability (33); f. Decreased T cell function. The extrinsic mechanisms include (27, 28): a. The inhibition of immunosuppressive cells in tumor microenvironment; b. There are other immune checkpoints on the surface of T cells, which will inhibit the function of T cells; c. The influence of gut microbiome (34).

## The Immunotherapy of HCC

### ICIs (Immune Checkpoint Inhibitors)

**Figure 1:**
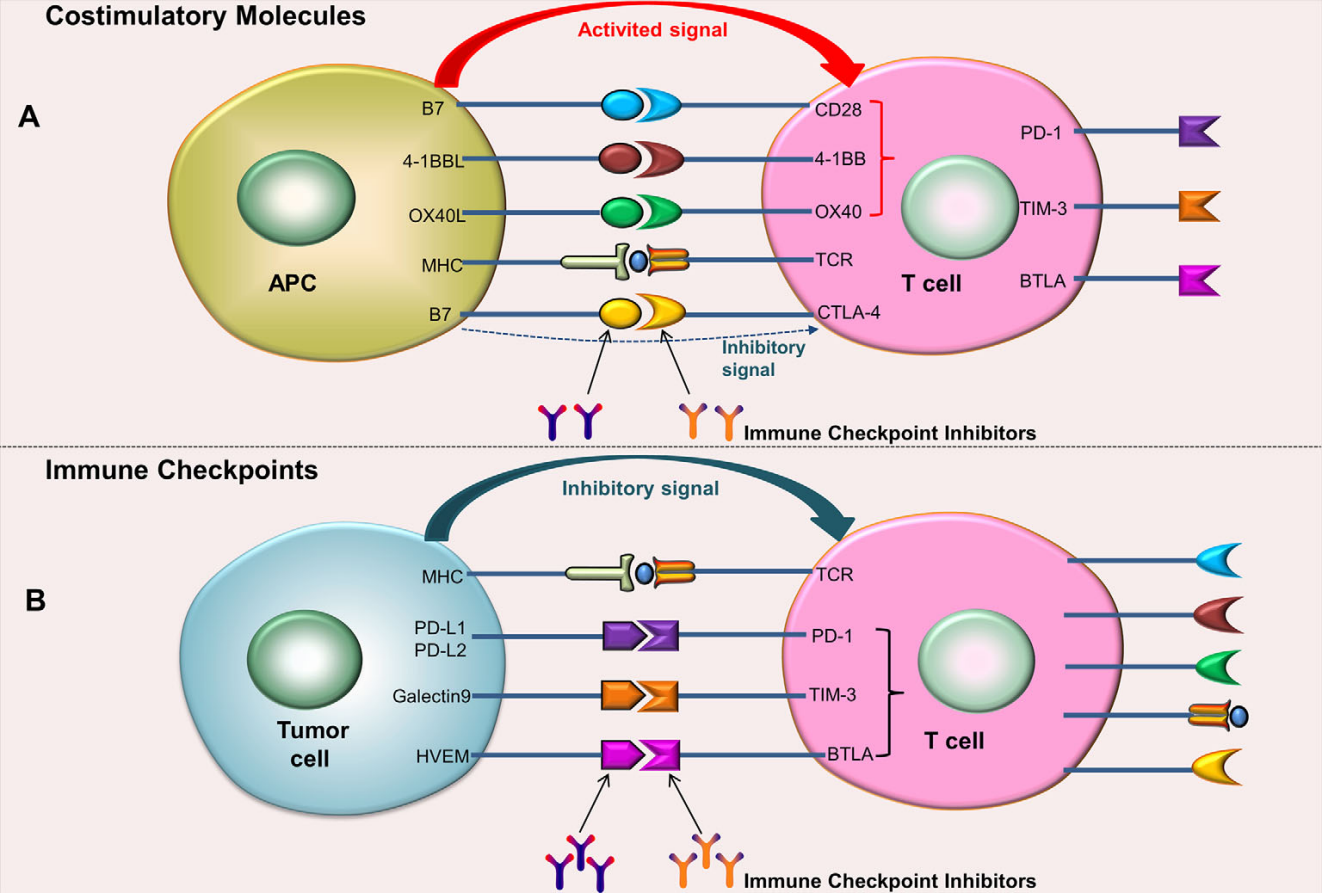
Costimulatory molecules and Immune checkpoints. **(A)** After the costimulatory molecules on the surface of T cells bind to their ligands (located on the surface of the APC), activation signals can be generated, which can promote the activation and proliferation of T cells; the combination of CTLA-4 and B7 produces an inhibitory signals. **(B)** After the immune checkpoints on the surface of T cells bind to their ligands (located on the surface of tumor cells), inhibitory signals can be generated, which can inhibit T cell activation and proliferation. ICIs can block immune checkpoints or their ligands, thereby blocking the transmission of inhibitory signals to T cells.

ICI is one of the most rapidly developing immunotherapy strategies nowadays. Immune checkpoints are membrane-bound molecules that can be expressed not only on the surface of many tumor cells but also on the surface of numerous immune cells (35). These immune checkpoints prevent and inhibit the activation of these cells through a physiological break (27). Immune checkpoints and costimulatory molecules are located on the surface of T cells, but their functions are opposite. Costimulatory molecules can provide activation signals (**Figure 1A**), while immune checkpoints provide inhibitory signals. When immune checkpoints bind to the ligands on the surface of tumor cells, the inhibitory signals transmitted from tumor cells can inhibit the activation and proliferation of T cells. The anti-tumor mechanism of ICIs is that they can block immune checkpoints or their ligands, thereby blocking the transmission of inhibitory signals to T cells (**Figure 1B**). The activation and proliferation of T cells with high expression of programmed cell death 1 (PD-1) are decreased; tumor cells with high expression of programmed cell death 1 ligand 1 (PD-L1)/programmed cell death 1 ligand 2 (PD-L2) are more likely to escape (36). Researchers analyzed 956 HCC samples and found that about 25% of the samples expressed high levels of PD-1 and PD-L1 (20). Common immune checkpoints include PD-1/PD-L1, cytotoxic T-lymphocyte antigen-4 (CTLA-4), T cell immunoglobulin-3 (TIM3), and Lymphocyte Activation Gene-3 (LAG3). For now, PD-1/PD-L1 and CTLA-4 are the most widely used.

#### PD-1/PD-L1 Inhibitors

PD-1 is a type I transmembrane glycoprotein expressed on the surface of most immune cells. These immune cells mainly include T cells, NK cells, regulatory cell (Treg), and myeloid-derived suppressor cell (MDSC) (37, 38). The main function of PD-1 is to negatively regulate the immune response. PD-L1 is the ligand of PD-1, which is mainly located on the surface of tumor cells. When PD-1 on the surface of T cells is combined with PD-L1 on the surface of tumor cells, tumor cells will transmit immunosuppressive signals to T cells. These inhibitory signals will inhibit the function of T cells and lead to T cell failure. Currently, the common PD-1 inhibitors include Nivolumab and Pembrolizumab, which are all-human IgG4 monoclonal antibodies. Common PD-L1 inhibitors include Durvalumab and Atezolizumab, which are IgG1 monoclonal antibodies.

In 2017, the United States Food and Drug Administration (FDA) approved Nivolumab for the treatment of HCC (39). In one clinical trial (40), 262 patients with advanced HCC received Nivolumab dose escalation and dose extension therapy. These patients included HCC patients who had previously received sorafenib or had not received sorafenib. For newly-treated patients who did not receive sorafenib, the objective response rate (ORR) after receiving nivolumab monotherapy was 20%~23%, and the media survival time was as long as 28.6 months; after receiving nivolumab monotherapy in patients who had received sorafenib, the ORR was 16%~19%, and the median survival time could reach 15.6 months (40). Another clinical trial showed that compared with sorafenib, patients receiving nivolumab did not achieve the expected overall survival (OS), but the OS rate, objective response rate (ORR), and complete remission (CR) rate were significantly improved (41).

Pembrolizumab is a humanized IgG4/kappa monoclonal antibody against IgG4/K, which can directly inhibit the binding of PD-1 to PD-L1. A phase II clinical trial in 2018 showed that 104 patients with HCC who were intolerant or still progressing after receiving sorafenib treatment were treated with Pembrolizumab (42). The results showed that the objective remission rate was 17%, 1% of the patients achieved complete remission, 44% of the patients were in stable condition, and 33% of the patients had disease progression.

For HCC patients, the high expression of PD-L1 is associated with lower tumor differentiation, a higher level of AFP, more frequent vascular invasion, and worse prognosis (43). PD-L1 inhibitors include Durvalumab and Atezolizumab. Atezolizumab has been used in the treatment of non-small cell lung cancer and triple-negative breast cancer. Presently, these inhibitors are in the evaluation stage of advanced HCC clinical trials.

#### CTLA-4 Inhibitors

CTLA-4 is a type of protein receptor located on the surface of T cells. CTLA-4 and CD28 receptors have two ligands in common: CD 80 and CD 86. Compared with CD28, CTLA-4 has a higher affinity with both ligands. CTLA-4-CD80 has the highest affinity, while CD28-CD86 has the lowest affinity (44, 45). CTLA-4 can compete with CD 28 for ligand binding, leading to a decrease in the co-stimulatory effect of CD 28 on T cells, and ultimately inhibiting T cell function (46). HCC is sensitive to CTLA-4 inhibitors (47). CTLA-4 inhibitors that have been approved by the FDA include Ipilimumab and Tremelimumab, etc. A phase II clinical trial showed that 20 patients with diagnosed advanced HCC were treated with Tremelimumab, ORR was 17%, disease remission rate (DCR) was 76.4%, and median progress free survival (PFS) was 6.48 months (48).

#### Combination Therapy of PD-1/PD-L1 Inhibitors and CTLA-4 Inhibitors

PD-1/PD-L1 and CTLA-4 pathways are different in negatively regulating immune activity, but their complementary effects are the same (47, 49). Blocking PD-1 or CTLA-4 can promote cell activation and proliferation, and alleviate immunosuppression mediated by Treg cells (50). Some pre-clinical studies of solid tumors showed that, compared with monotherapy, the combination of PD-1 inhibitor and CTLA-4 inhibitor could produce synergistic effects and enhance their anti-tumor activity (51). The Results of The CheckMate 040 Randomized Clinical Trial in 2020 showed that the ORR and DCR of Nivolumab combined with Ipilimumab in the treatment of advanced HCC were 32% and 27%, respectively (47). This study proved that the combination of two immunosuppressants might have a better therapeutic effect and was also safe for HCC patients. Furthermore, increasing the dose of Ipilimumab might improve the persistent response and prolonged the survival time of patients with advanced HCC. The NCT02519348 study (52) also showed that the combination of tremelimumab and durvalumab was more effective than single drug [ORR: 22.7% *VS* (7.2% and 9.6%)] and had an encouraging safety. ICIs can also be combined with other treatment strategies, these strategies include locoregional treatments, antiangiogenetic therapy, chemotherapy, the mammalian target of rapamycin inhibitor, etc. (53).

Immune checkpoint inhibitors represented by anti-PD-1/PD-L1 and anti-CTLA-4 antibodies have shown good results in the clinical treatment of HCC, providing a new treatment method for HCC patients (**Table 1**). Nevertheless, the safety, efficacy, and prognosis of the combination of 2 ICIs still require extensive clinical studies to verify.

**Table 1:** Partial research results of ICIs for HCC.

Medicine	Time	Case	Test Phase	OS (month)	Median PFS (month)	ORR (%)	DCR (%)	Trial Registration
**Anti-PD-1**								
Nivolumab	2017	214	I/II	15.1	4	20	64	NCT01658878 (40)
Nivolumab	2021	49	I/II	NO	NO	12	55	NCT01658878 (54)
Pembrolizumab	2018	104	II	12.9	4.9	17	62	NCT02702414 (42)
(Pembrolizumab/placebo)	2020	278/135	III	13.9/10.6	3.0/2.8	18.3/4.4	62.2/53.3	NCT02702401 (55)
Camrelizumab	2020	217	II	13.8	2.1	14.7	44.2	NCT02989922 (56)
**Anti-PD-L1**								
Durvalumab	2019	39	I/II	13.2	2.7	10.3	33	NCT01693562 (57)
**Anti-CTLA-4**								
Tremelimumab	2013	20	II	8.2	6.5	17.6	76.4	NCT01008358 (48)
**Anti-PD-1/PD-L1 + Anti-CTLA-4**								
(Nivolumab + Ipilimumab)	2020	148	I/II	NO	NO	32	27	NCT01658878 (47)
(Durvalumab + Tremelimumab)	2020	332	III	NO	NO	22.7	NO	NCT02519348 (52)
(Ipilimumab + Nivolumab/pembrolizumab)	2021	25	I	NO	NO	16	40	N.F. (58)

OS, overall survival; PFS, progression-free survival; ORR, objective remission rate; DCR, disease control rate.

N.F., related information not found.

#### ICIs Combined With Other Therapies for HCC

Although ICIs have achieved certain clinical efficacy, it is necessary to adopt some combination strategies to further improve its efficacy due to the limited response rate of monotherapy. These strategies include combined molecular targeted drugs, combined chemotherapy, combined radiotherapy, combined TACE and combined ablation.

##### ICIs Combined With Molecular Targeted Drugs

Molecular targeted therapy is to block or inhibit the key genes or signal pathways in the process of tumor occurrence and development at the molecular level, and finally achieve the purpose of anti-tumor. Sorafenib, a multiple tyrosine kinase inhibitor (TKI), is the first molecular targeted drug approved for the treatment of advanced HCC. TKI mainly achieves the purpose of anti-tumor by inhibiting the tyrosine kinases of several growth factor receptors. It has been proven that sorafenib can only prolong the survival time of HCC patients by several months. Consequently, it is necessary to develop a combined therapy to further improve the clinical efficacy.

Lavatinib, a TKI, has become the first-line treatment for advanced HCC. Finn et al. (59) combined Pembrolizumab (PD-1 inhibitor) with lenvatinib (a multikinase inhibitor) to treat unresectable HCC (uHCC). Test results were evaluated with modified Response Evaluation Criteria In Solid Tumors (mRECIST). The results showed that ORR was 46%, DCR was 88%, median PFS was 9.3 months, median DOR was 8.6 months, and median OS was 22 months. In addition, 67% of patients had treatment-related adverse events (≥Grade 3). The experiment conclusion: Lenvatinib + Pembrolizumab had a good anti-tumor activity against uHCC, and its safety was acceptable. The study by Llovet et al. (60) also showed that Lenvatinib + Pembrolizumab had an encouraging effect for uHCC patients.

In another phase III clinical trial of ICIs combined with targeted drugs to treat uHCC, Finn et al. (61) enrolled a total of 501 uHCC patients. The experimental group (336) was treated with Atezolizumab (PD-L1 inhibitor) + Bevacizumab (anti-angiogenesis); the control group (165) was treated with Sorafenib monotherapy. The experimental results (mRECIST) showed that OS and PFS of the experimental group were significantly better than those of the control group, and the incidence of adverse events between the two groups had no significant difference (98.2% *VS* 98.7%). Currently, Atezolizumab+ Bevacizumab has become the first-line treatment for patients with advanced HCC (62). ICIs combined with molecularly targeted drugs may have a synergistic effect (63, 64) and have promising prospects in the treatment of advanced HCC. These synergistic effects include (65): a. Targeted drugs themselves have anti-tumor effects; b. Targeted drugs can improve DC (dendritic cell) activation and immune cell infiltration; c. Targeted drugs can block the PD-1/PD-L1 pathway; d. Combination therapy can affect Wnt/β-catenin activated mutations.

##### ICIs Combined With Chemotherapy

Although ICIs are effective in the treatment of many immunogenic tumors, for those cold tumors, ICIs are ineffective in most cases (66). Chemotherapy drugs can inhibit or kill tumor cells, and the destroyed tumor cells can release tumor-related antigens, which can stimulate the body to produce an immune response. Besides, chemotherapy can also consume immunosuppressive cells (such as MDSCs and Tregs) to reduce or relieve the immunosuppressive effect of tumor microenvironment (TME) (67). In the past, chemotherapy was considered to have only immunosuppressive effects, but recently, some new viewpoints suggest that chemotherapy may also have immunostimulatory effects (66) and participate in the active regulation of the immune system (which can transform cold tumors into hot tumors) (68). In a phase II clinical trial evaluating Camrelizumab+FOLFOX4 in the treatment of advanced HCC (69), the researchers included 34 patients with advanced HCC. The experimental results (RECIST) showed that ORR was 26.5%, DCR was 79.4%, and median PFS was 5.5 months. Meanwhile, this combination therapy is tolerable for patients with advanced HCC. ICIs + chemotherapy may provide a promising option for the treatment of patients with advanced HCC.

##### ICIs Combined With Radiotherapy (RT)

As one of the most important cancer treatment methods, the basic principle of RT is to use high-energy particles to induce DNA damage in tumor cells, which eventually leads to tumor cell death. In recent years, it has been found that RT can not only kill tumor cells directly, but also induce immune-related anti-tumor responses (70). The mechanism mainly includes: a. RT can induce tumor cell death to release large amounts of tumor-associated antigens. These antigens can stimulate the body to produce an immune response; b. RT can up-regulate the expression of major histocompatibility complex class I (MHC-I) molecules, allowing CD8+ T cells to recognize and kill tumor cells (71); c. RT can increase the number of tumor-infiltrating lymphocytes (TILs) in tumor tissue (70); d. RT can improve the immunogenicity of tumor cells, and at the same time, it can also cause immunosuppression. The study of Chew et al. (72) showed that RT could increase the expression of PD-1 and Tim-3 on the surface of CD8+ T cells. Apart from that, RT can also increase the expression of PD-L1 on the surface of tumor cells (73, 74). The role of ICIs is to block these immune checkpoints. Accordingly, the combination of ICIs and RT can produce synergistic effects (70, 75). One preclinical study by Kim et al. (74) showed that compared with anti-PD-L1 therapy or RT alone, the combination of the two methods can significantly improve the anti-tumor ability and the survival rate. Chiang et al. (76) treated 5 uHCC patients with stereotactic body radiotherapy (SBRT) + Nivolumab. The experimental results showed that ORR could reach 100%, 2 patients got complete remission (CR), 3 patients got part remission (PR), and mPFS reached 14.9 months. Additionally, only 1 patient had ≥3 Grade adverse reactions. For now, there are few clinical trial data about ICIs combined with RT in the treatment of HCC. The best combination therapy for HCC still needs to be explored.

##### ICIs Combined With Transarterial Chemoembolization (TACE)

TACE was first proposed and applied in clinic in 1977. TACE belongs to palliative treatment, and in most cases it cannot achieve radical cure. Its mechanism of action is to deliver chemotherapeutic drugs to the hepatic artery to embolize the artery, causing ischemic necrosis of tumor tissue; in the meanwhile, chemotherapeutic drugs also play an anti-tumor effect. Due to the rich blood supply of the liver, the portal vein will still supply blood to the tumor tissue after the artery is embolized. As a result, patients with HCC who undergo TACE tend to have a high rate of postoperative recurrence (77). In a phase I clinical trial of ICIs combined with TACE in the treatment of uHCC (78), a total of 9 uHCC patients received Nivolumab + drug eluting bead transarterial chemoembolization (deb-TACE). The results of the study showed that DCR reached 100% (PR was 22%, SD was 78%), and 12-month OS rate was 71%; as an aside, this combination therapy was safe and tolerable. Another phase I clinical trial evaluating Pembromizumab combined with TACE in the treatment of advanced HCC also showed that Pembrolizumab+TACE had tolerable safety with no synergistic toxicity (OS, PFS, ORR and DCR had not been released yet) (79). Up to now, although there are few clinical experimental data of ICIs+TACE in the treatment of HCC, its future development prospect is promising.

##### ICIs Combined With Ablation

Tumor ablation is one of the main interventional treatments for HCC. It mainly includes radiofrequency ablation (RFA), microwave ablation (MWA) and cryoablation. Both RFA and WMA deliver large amounts of energy to the tumor site, leading to local heating and destroying tumor cells through thermal efficiency. Cryoablation is to freeze tumor tissue at local low temperature, which induces delayed necrosis of tumor cells after injury.

Ablation can also mediate immune regulation (80). The mechanism may include: a. Tumor-associated antigens released after death of tumor cells can activate adaptive immune cells (81); b. Following RFA or MWA, large quantities of (Heat shock proteins-70) HSP-70 are released in serum, which may lead to local inflammation and activation of antigen-presenting cells (APCs) in tumor area, thus inducing anti-tumor response (82). c. RFA increases the infiltration of dendritic cells (DCs) in tumor tissues and significantly enhances the response of CD4+ T cells and CD8+ T cells (83). d. After receiving RFA locally, the number of central memory lymphocytes increased remarkably (84). e. After receiving RFA locally, the expression of inhibitory cytokines decreased and the level of anti-tumor cytokines increased (81). At the present time, there are few studies about the effect of MWA and cryoablation on tumor immunity of HCC patients. The study of Leuchte et al. (85) showed that MWA can enhance the tumor-specific immune response of HCC patients. In a I/II clinical trial, a total of 32 patients with advanced HCC received Tremelimumab + (RFA/chemoablation) (86). The experimental results showed that 5 (26.3%) of the 19 evaluable patients achieved PR; median time to tumor progression (TIP) was 7.4 months; median OS was 12.3 months; 6-month tumor PFS and 12-month tumor PFS were 57.1% and 33.1%, respectively; and no dose limiting toxicity occurred in this trial. As of now, there are few clinical experimental data about ICIs combined with ablation in the treatment of advanced HCC, and a large number of clinical experimental data are still needed to explore the best combination scheme. ICIs and ablation have different anti-tumor mechanisms, which may produce synergistic effects in the combined treatment of tumors. In conclusion, ICIs combined with other therapies is an effective and potential treatment for HCC (**Table 2**).

**Table 2:** Partial research results of ICIs combined with other therapies to treat HCC .

Medicine	Time	Case	Test Phase	OS (month)	Median PFS (month)	ORR (%)	DCR (%)	Trial Registration
Anti-PD-L1+ molecular targeted drugs								
(Durvalumab + Ramucirumab)	2020	28	Ia/b	18	4.4	11	61	NCT02572687 (87)
(Atezolizumab + Bevacizumab/Sorafenib)	2020	336/165	III	NO	6.8/4.3	27.3/11.9	73.6/55.3	NCT03434379 (61)
(Avelumab + axitinib)	2019	22	Ib	NO	3.8	31.8	NO	NCT03289533 (88)
(Lenvatinib + pembrolizumab)	2020	104	I	22	9.3	46	88	NCT03006926 (59)
(Lenvatinib + pembrolizumab)	2019	67	I	NO	NO	44.8	82.1	NCT03006926 (60)
Anti-PD-1+ molecular targeted drugs								
(Sintilimab+ IBI305, high-dose/low-dose)	2020	21/29	Ib	NO	NO	33.3/24.1	83.3/N.F.	NCT04072679 (89)
Anti-PD-1+ chemotherapy								
(Camrelizumab+ FOLFOX4)	2019	34	II	NO	5.5	26.5	79.4	NCT03092895 (69)
(Lenvatinib+ FOLFOX4)	2020	24	I	NO	NO	66.7	79.2	N.F. (90)
Anti-PD-1+ RT(Nivolumab+RT)	2019	5	I	NO	14.9	100	100	N.F. (76)
Anti-PD-1+ TACE(Nivolumab+ deb-TACE)	2020	9	I	NO	NO	100	78	NCT03143270 (78)

IBI305, a biosimilar candidate of bevacizumab; N.F., related information not found; FOLFOX4, infusional fluorouracil, leucovorin and oxaliplatin; TACE, transarterial chemoembolization; RT, radiotherapy.

### Tumor Vaccines

The principle of tumor vaccines is to introduce tumor antigens into patients in various forms, so as to overcome the immunosuppression caused by tumor, enhance the immunogenicity of tumor cells, activate the immune system of patients, and eventually achieve the purpose of anti-tumor. For the moment, tumor vaccines used for HCC treatment and research mainly include nucleic acid vaccines, peptide vaccines, oncolytic virus vaccines, and DC vaccines.

#### Nucleic Acid Vaccines

**Figure 2:**
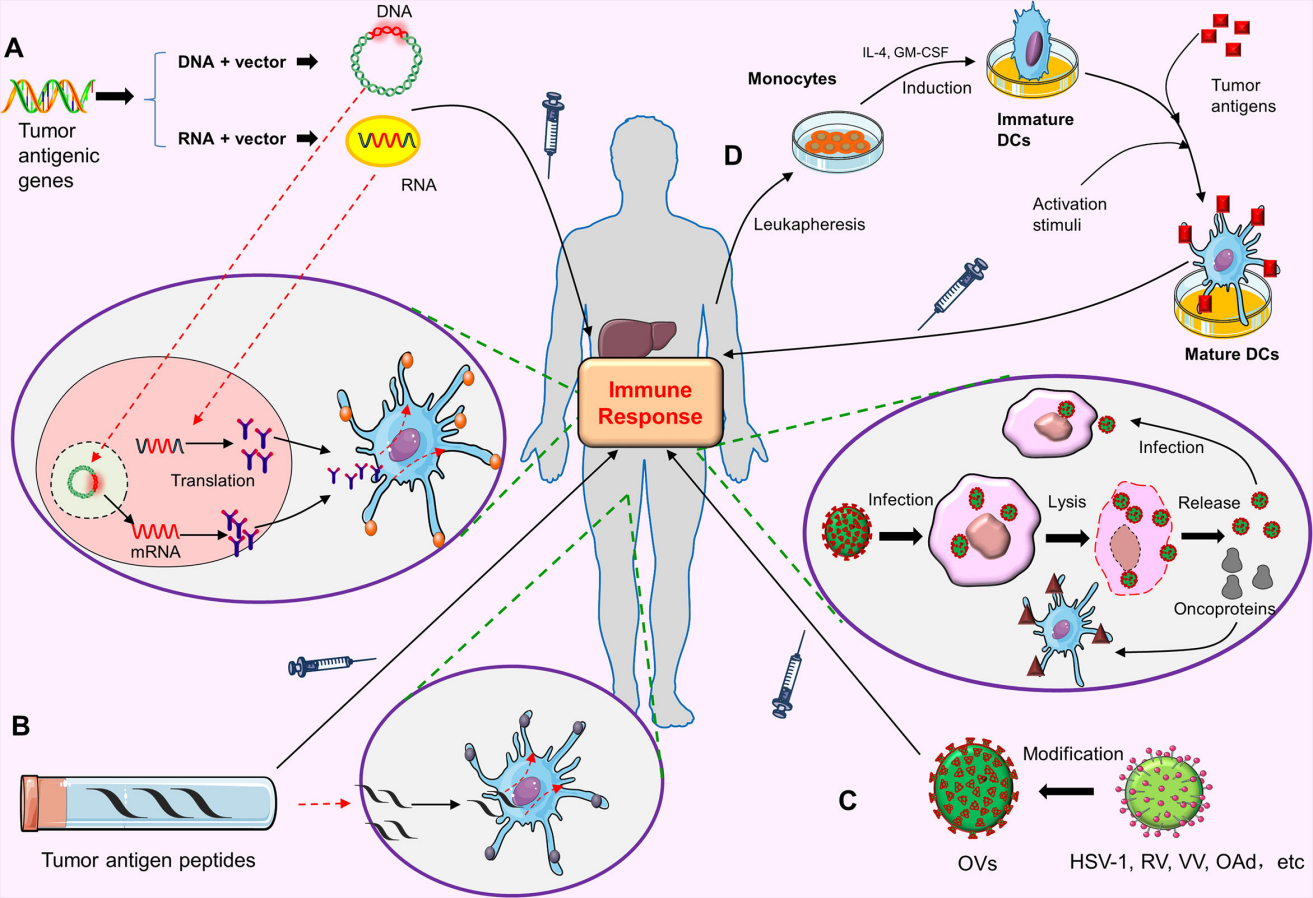
The preparation process and anti-tumor mechanism of tumor vaccines. **(A)** Nucleic acid vaccines. The gene (DNA or RNA) encoding a tumor antigen is recombined with the vector and injected into the patient. DNA needs to enter the nucleus of the host cells, while RNA only needs to enter the cytoplasm of the host cells to be translated into the corresponding proteins. These proteins are secreted out of cell, captured by APCs, and finally activate the human immune response to this tumor antigen. **(B)** Peptide vaccines. High-dose tumor antigen peptides are delivered to the MHC molecules on APC surface, thus stimulating the specific immune response of the body to this tumor antigen. **(C)** Oncolytic viruses(Ovs). OVs can be modified from HSV-1, RV, VV and OAd. OVs can infect tumor cells after entering the body, and proliferate in large amounts in tumor cells, eventually leading to tumor cell lysis and death. Dead tumor cells release OVs virus and tumor proteins. The released OVs virus can continue to infect other tumor cells. Tumor proteins can be captured by APCs and eventually activate the host immune response. **(D)** DC vaccines. Monocytes are extracted from the blood of patients. These monocytes are induced to become Immature DCs under the stimulation of IL-4 and GM-CSF. Then under the action of activation stimuli, the tumor antigens are loaded on the Mature DCs. Finally, these DCs loaded with tumor antigens are injected into the body to cause the body to produce the immune response to tumor cells.

Nucleic acid vaccine refers to the recombination of a gene (DNA or RNA) encoding a certain tumor antigen with a vector, and then injecting it into the patient. After these nucleic acids enter the host cells, the host cells can express the corresponding polypeptides or proteins, thus inducing the body to produce an immune response against these antigens (**Figure 2A**). DNA vaccines are easy to manufacture, low cost, and stable. Unfortunately, DNA cannot be amplified in transfected cells like viral vectors. It needs to enter the nucleus to be translated into the corresponding proteins. Moreover, it is not easily taken up or expressed by DC cells, therefore, it cannot induce the body to produce an effective immune response (91). Once the DNA is integrated into the genome, there may be such a risk, that is, causing the activation of oncogenes or the inactivation of tumor-suppressor genes. In contrast, RNA vaccines are safer than DNA vaccines. RNA can participate in the synthesis of protein only by entering cytoplasm, but cannot be integrated into the genome. The potential carcinogenicity is weaker. Nevertheless, the poor stability and short half-life of RNA vaccine limit its clinical application to a certain extent. Up until now there are few clinical trials of nucleic acid vaccines for HCC, and most of them are still in preclinical research stage.

#### Peptide Vaccines

Tumor antigen peptides are important components of peptide vaccines. Tumor antigens must be degraded into short peptides and form peptide-MHC-TCR complex in antigen-presenting cells (APCs) to be recognized by T cells and stimulate the corresponding cytotoxic T lymphocyte (CTL) response. The purpose of peptide vaccines is to deliver a high-dose tumor antigen peptides to major histocompatibility complex (MHC) molecules on the surface of APCs, so as to stimulate the specific immune response of the body to tumor cells (**Figure 2B**).

There seem AFP peptide vaccine and GPC3 peptide vaccine which are studied more frequently nowadays. Alpha-fetoprotein (AFP) is one of the most common serum markers in the diagnosis of HCC, and its high expression in hepatocellular carcinoma cells makes it a promising target for vaccine-based therapy (92). AFP peptide vaccine showed good anti-tumor activity in the treatment of HCC (93, 94). One clinical research of glypican-3 (GPC3) peptide vaccine in the treatment of HCC patients revealed that GPC3 peptide vaccine had good tolerance and anti-tumor effect, as well as could prolong the overall survival time of patients (95). Although plenty of tumor antigens have been found in liver cancer, only the vaccines targeting AFP, GPC3 and MRP3 show good tolerance and safety, and the specific T cell response rate of these vaccines exceeds 70% (96).

#### Oncolytic Viruses(OVs)

Oncolytic viruses are a type of viruses that can effectively infect and kill cancer cells. When the virus-infected cancer cells rupture and die, the newly generated virus particles will be released to further infect the surrounding cancer cells. Ovs can not only directly kill tumor cells but also stimulate the immune response of the body and enhance the anti-tumor effect (**Figure 2C**). Most Ovs are modified from herpes simplex virus-1 (HSV-1), reovirus (RV), Vaccinia virus (VV) and oncolytic adenovirus (OAd). OVs can be designed or screened to selectively amplify and kill cancer cells in cancer cells. They can play a role in the primary tumors, as well as in the metastatic tumors (97). A general design principle is to weaken or delete viral virulence factors, and prevent OVs from replicating in normal cells by using tumor-specific distortion of signal pathway in cancer cells, but they can still maintain replication and killing activity in cancer cells (97). JX-59 (pexastimogene devacirepvec, Pexa-Vec) is one of the most commonly used oncolytic viruses (modified from VV) in HCC-related clinical trials. It can be replicated preferentially in cancer cells. The results of one clinical trial showed that it had good safety and could improve the overall survival rate of patients with unresectable HCC (98). Phase I and II clinical trials of advanced HCC related to JX-594 are ongoing (for example, NCT 01636284 and NCT 03071094) at the present time.

#### DC Vaccines

Dendritic cells (DCs) are the most powerful antigen presenting cells (APCs) so far. They are named because they mature with many dendritic or pseudopod-like protrusions. DCs have the function of immune response and immune tolerance, which is of great significance for maintaining immune balance. There exit many inhibitory cytokines in the tumor microenvironment. These inhibitory cytokines can inhibit the normal function of DCs and promote the escape of tumor cells (99). DC vaccine is the focus of tumor immunotherapy in recent years. The principle is to load tumor antigens on DCs, and then inject these DCs loaded with tumor antigens into the body; these DC cells can promote the proliferation of cytotoxic T lymphocytes (CTL) *in vivo*, and ultimately play the role of anti-tumor (**Figure 2D**). In terms of basic research and clinical application, DC vaccines have shown the application prospects in tumor prevention and tumor treatment (100, 101).

In the phase I and II clinical studies of the DC vaccines in the treatment of patients with advanced HCC, the results showed that the DC vaccines had the tumor-specific immune responses in patients with advanced HCC (102, 103). In a phase II clinical trial, 39 patients with advanced HCC received DC vaccine treatment, of which 25 patients were evaluable with a disease control rate (DCR) of 28% (102). Moreover, all the subjects had no grade 3-4 adverse reactions. The results suggest that the DC vaccine was effective and safe in patients with HCC. Another study (104) showed that patients with HCC were treated with transcatheter arterial embolization (TAE) or TAE combined with DC vaccine, and the results showed that the combined treatment group could more effectively enhance tumor-specific immune response. However, there was no difference in tumor recurrence rates between the two groups, which might be related to the immunosuppressive microenvironment of HCC and the lack of specific HCC target antigens in DC vaccines. This may be related to the immunosuppressive microenvironment of HCC and the lack of specific HCC target antigens in current DC vaccines. About 10% of tumor antigens have immunogenicity, and only a few are tumor rejection antigens, which can trigger immune responses and kill tumors. If these tumor rejection antigens can be fully utilized when constructing DC vaccines, it may further enhance the anti-tumor effect of DC vaccines.

It is also very important for DC vaccine to choose the appropriate route of administration. Presently, the main routes of administration include intravenous, subcutaneous and intradermal routes. Optimizing the route of administration is conducive to more effective vaccination. The intradermal route of DC vaccination shows that the migration level of DCs to the lymph nodes is very low; the ultrasound-guided intra-lymph node vaccination of DC vaccines has the risk of injecting the vaccine into fat rather than a cellular area (105). DCs may not be able to reach the tumor site effectively. Therefore, DCs need to be further improved to increase the ability to migrate to the tumor; or change the way of receiving DC vaccines to increase the number of DCs in tumors. For example, *in situ* DC vaccination, that is, direct intratumoral inoculation of unloaded DCs produced *in vitro*. The unloaded DCs can uptake a variety of TAAs in the tumor, so there is no need to generate TAA vectors *in vivo* and select specific targets (106). Furthermore, the combination of *in situ* DC vaccine and immunogenic cell death (ICD) inducer can further improve the antitumor effect of DCs. Because ICD inducers can not only cause tumor cell death and release TAAs but also increase the secretion of damage-associated molecular patterns (DAMPs) that can activate DCs (107–109).

DC vaccine is a safe and promising anti-tumor therapy (110). Although DC vaccine as an independent therapeutic agent may have limitations, combined with other treatments can improve the effectiveness of treatment. Zhou et al. (111) found that compared with advanced HCC patients who only received sorafenib, dendritic cells and cytokine-induced killers (DC-CIK) combined with sorafenib could increase the tumor response rate and prolong OS of patients without increasing the incidence of adverse events.

### Adoptive Cell Therapy (ACT)

ACT is an immunotherapy based on the use of autoimmune cells of cancer patients. The main process is to isolate the immunocompetent cells in the tumor patient, modify these immune cells or stimulate them with some cytokines. These immune cells are amplified and screened *in vitro*, and then they are returned to the patient. ATC achieves the purpose of anti-tumor by enhancing the immune function of patients or targeting to kill tumor cells. Commonly used ACT includes natural killer (NK) cells, cytokine-induced killer (CIK) cells and chimeric antigen receptor (CAR)-T cells, etc.

#### NK Cells

NK cells are one of the most important immune cells in human innate immunity, which can produce non-specific immune responses without being sensitized by antigens. The activation state of NK cells is determined by the dynamic balance of the expression of inhibitory receptors and activated receptors on the surface of NK cells. NK cells account for 30% ~ 50% of innate immune cells in the liver (112), which are responsible for presenting cytotoxic granules, secreting effector cytokines, and cooperating with apoptotic receptors to induce apoptosis of target cells.

NK cells in cancer patients are often in a state of functional failure due to the immunosuppressive effect of the tumor microenvironment (TME). If the failure state of these NK cells can be reversed, it is possible to restore their anti-tumor effect. One animal study (113) showed that Sirtuin2 (SIRT2) could reactivate the anti-tumor activity of depleted NK cells in hepatoma mice. SIRT2 could significantly promote the production of cytokines and cytotoxic mediators by activated NK cells. Similarly, NK cells overexpressing SIRT2 showed a stronger antitumor effect on hepatoma cells. Consequently, it is possible to improve the prognosis of HCC patients by adding NK cells with anti-tumor activity to reshape the immune system of the liver.

NK cells come from a wide range of sources, have a broad-spectrum oncolytic effect, and are not restricted by MHC. According to the source of NK cells, NK cell adoptive immunotherapy can be divided into autologous NK cell immunotherapy and allogeneic NK cell immunotherapy. Autologous NK adoptive immune cells are obtained by stimulating the *in vitro* expansion of CD56^Birght^ NK cells in peripheral monocytes with cytokines. NK cells in peripheral blood can proliferate 140 times in a short time by stimulating with some cytokines (114). Meanwhile, these amplified and activated NK cells show strong anti-tumor effects *in vitro* and *in vivo* (114). Nonetheless, the clinical efficacy of autologous NK cells is not significant (115). The high expression of inhibitory killer cell immunoglobulin-like receptor (KIR) on the surface of tumor cells can inhibit NK cell functions after binding to NK cells, which makes tumor cells prone to immune escape. Allogeneic NK cells that do not match the KIR on the surface of tumor cells have better clinical efficacy (116). One clinical study showed that the use of allogeneic NK cells could improve the immune function of HCC patients in a short period of time (117). How to further improve the accuracy and persistence of NK cells in the future is still the focus of research.

#### Cytokine-Induced Killer (CIK) Cells

CIK cells refers to the heterogeneous cell population mainly consisting of CD3+CD8+ and CD3+CD56+, which are formed by co-culturing peripheral blood mononuclear cells with cytokines (such as IL-1, IL-2 and IFN-γ). Its mechanism of action is to directly kill tumor cells by releasing perforin and granzyme, or indirectly by releasing a variety of cytokines. Similarly, it can also induce tumor cell apoptosis by activating apoptotic genes. CIK cells mainly include NK-like T lymphocytes (NKT cells) and cytotoxic T lymphocytes. Among them, NKT cells are the main effector cells that exert anti-tumor effects.

A retrospective study showed that CIK could significantly improve OS of HCC patients (118). The results of another phase III clinical trial showed that CIK cell therapy could prolong the progression-free survival (PFS) of HCC patients to 44 months (119). The 5-year follow-up results showed that compared with the control group, PFS and OS of HCC patients (receiving CIK treatment group) were significantly prolonged (120). On the contrary, some researchers believed that CIK therapy might not significantly improve OS of HCC patients (121). Additionally, the researchers found that CIK cells could increase the infiltration of immunosuppressive cells in tumors, thus inhibiting their anti-tumor activity (122). Although the long-term curative effect of CIK cell therapy for HCC patients still needs numerous clinical studies to prove, CIK cell therapy is still a promising immunotherapy for HCC patients.

#### T Cell Receptor (TCR)-T Cell Therapy

TCR is a specific receptor on the surface of T cells. It activates T cells by recognizing and binding antigens presented by MHC, and ultimately promotes the differentiation and proliferation of T cells. The principle of TCR-T cell therapy is to introduce TCR genes that specifically recognize tumor antigens into patients’ T cells by gene editing technology, so that these T cells can express corresponding TCR on their surfaces. Then these TCR-T cells are screened and amplified *in vitro*, and finally injected into patients. TCR-T cell therapy can effectively identify and kill tumor cells by enhancing the specific recognition ability of T cells to tumor cells and improving the affinity of T lymphocytes to tumor cells. Compared with CAR-T cells, TCR-T cells can not only recognize antigens on the surface of tumor cells but also intracellular antigens. Currently, some phase I and II clinical trials exploring TCR-T cell treatment of HCC (for example, NCT01967823, NCT03132792, NCT02719782, etc.) are in progress.

#### (Chimeric Antigen Receptor) CAR-T Cell Therapy

In recent years, CAR-T cell therapy has become a research hotspot in adoptive cell therapy. CAR is mainly composed of four parts, including, single chain variable fragment (scFv); hinge region; transmembrane region (TM); intracellular signal domain (immunoreceptor tyrosine-based activation motif, ITAM). The principle is to construct CAR genes that recognize tumor antigens *in vitro* and combine them with vectors to form recombinant plasmids. Then transfecting these plasmids into patient T cells, which makes these T cells express the corresponding CARs. These CAR-T cells are screened and expanded *in vitro*, and finally returned to the patient. They can target to kill the corresponding tumor cells after entering the body. Compared with TCR-T cells, CAR-T cells do not require antigen processing and MHC presentation.

**Figure 3:**
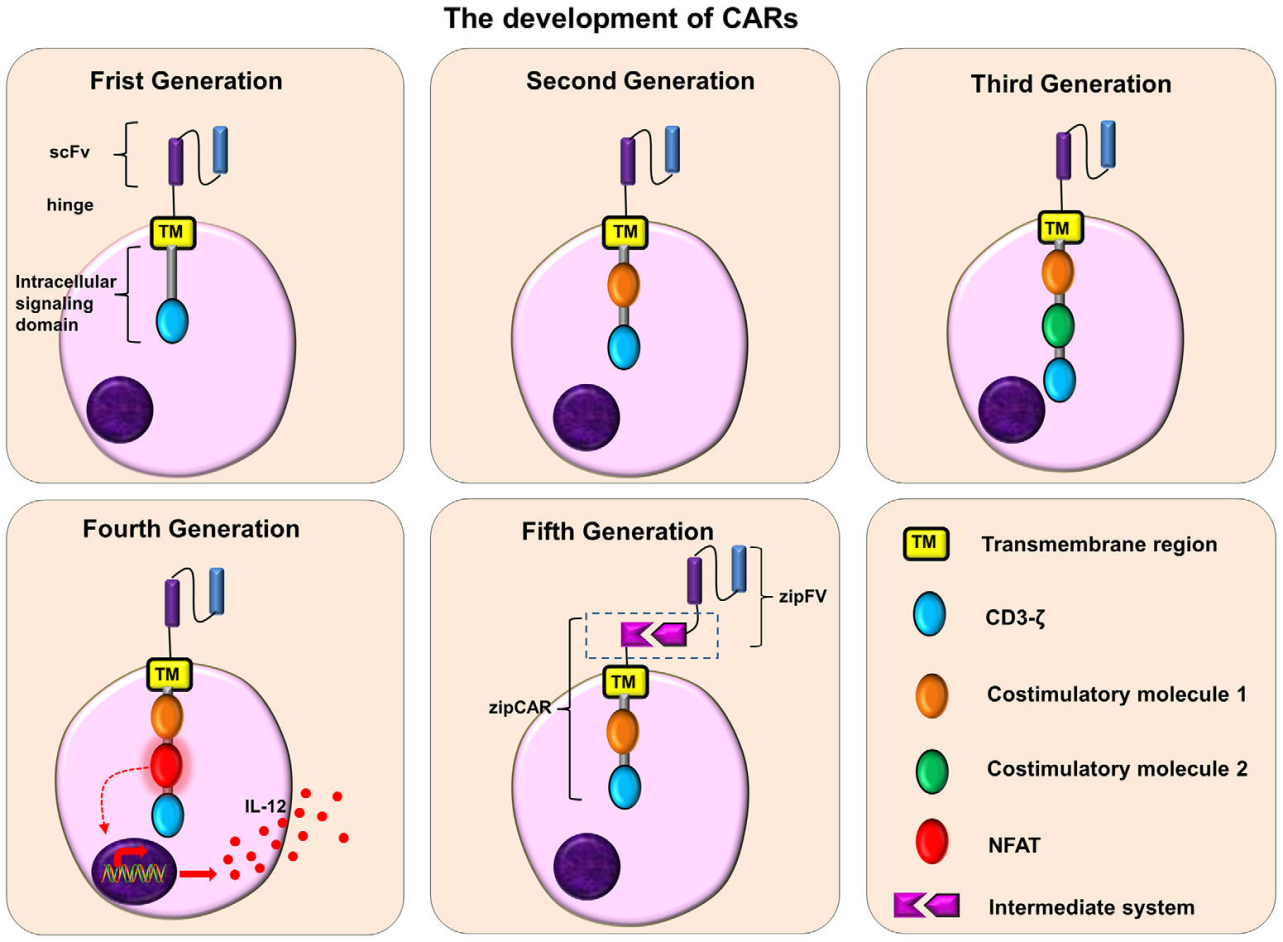
The development of CARs. Frist Generation: scFv + TM + CD3ζ; Second Generation: scFv + TM + one costimulatory molecule + CD3ζ; Third Generation: scFv + TM + two costimulatory molecule + CD3ζ; Fourth Generation: scFv + TM + one/two costimulatory molecule + NFAT + CD3ζ; Fifth Generation: scFv+ intermediate system + one/two costimulatory molecule + CD3ζ.

Since it was put forward by CAR in 1989, it has developed from the first generation to the fifth generation (**Figure 3**). The intracellular domain of the first-generation CARs has only one signal domain (CD3 ζ). Due to the lack of costimulatory molecules, the CAR-T cells can recognize the corresponding tumor cells, but the clinical effect is limited (123). The main reason for this phenomenon is that the first-generation CAR-T cells have poor persistence in the body, and the proliferation of CAR-T cells is low, which eventually leads to CAR-T cells apoptosis.

The second-generation CARs add a costimulatory molecule to the first-generation CARs, such as CD28, 4-1BB (CD137), OX40 (CD134) or ICOS, etc. These costimulatory factors can promote the proliferation of CAR-T cells, the secretion of cytokines, and the secretion of anti-apoptotic proteins, thus improving the persistence of CAR-T cells and their ability to kill tumors. Among them, the CAR with CD 28 or 4-1 BB has a stronger tumor-killing effect (124). Furthermore, compared with CAR-T cells containing CD28, CAR-T cells containing 4-1BB are more durable and may be more resistant to exhaustion (125, 126).

The third-generation CARs contain two costimulatory molecules, which further enhances the activation, proliferation and persistence of T cells, and makes CAR-T cells have a stronger tumor-killing effect. Moreover, the third-generation CARs that contain both CD 28 and 4-1 BB can provide the strongest anti-tumor effect (127).

The fourth-generation CARs, also known as “TRUCKs”, are modified by adding nuclear factor of activated T cells (NFAT) on the basis of the second or third-generation CARs. For example, the most common NFAT is the cytokine IL-12. These fourth-generation CAR-T cells (IL-12) can release IL-12 after being activated, which can not only promote T cell activation and regulate immunity, but also recruit other innate immune cells to attack tumor cells (128–131). In order to reduce the toxicity associated with CAR-T cells, some fourth-generation CARs have added suicide genes. When necessary, the suicide gene system can induce CAR-T cell death or shorten its lifespan (132, 133).

The fifth-generation of CARs, are also called “universal CARs”. Theoretically, these CARs can target different tumor antigens. In traditional CAR-T cell therapy, one type of CAR-T cells can only target one kind of tumor surface antigens. In order to improve the flexibility and controllability of CARs and expand the scope of antigen recognition, the fifth-generation CAR adopts a “third-party” intermediate system to separate the antigen binding domain of CAR from its T cell signal unit (131, 134, 135). These CAR-T cells can target different tumor antigens to fight against tumor heterogeneity, and can also improve their safety and reduce related toxicity during the treatment of CAR-T cells (135). The common “Third-party” intermediate systems are biotin-binding immune receptors (BBIR) CAR (136) and programmable (SUPRA)CAR (137).

CAR-T cell therapy has made great achievements in the treatment of hematologic malignancies. It also has great potential in the treatment of solid tumors. One of the key factors affecting the curative effect of CAR-T cells is the selection of tumor surface antigens. CAR-T cells are designed for one or more tumor antigens, so they can specifically identify tumor cells expressing these tumor antigens. For CAR-T cells, the best design scheme should be to use tumor-specific antigens (TSAs) to design the corresponding CARs, because these CARs are more targeted and can minimize off-target effects. Unfortunately, there seem too few TSAs, and most of the common targets are tumor-associated antigens (TAAs). Currently, there are some therapeutic targets of CAR-T cells for HCC research and treatment (some HCC-related CAR-T cell therapy targets, **Table 3**). Among them, phosphatidylinositol proteoglycan 3 (GPC3) is the most widely used. GPC3 is a membrane protein located on the surface of tumor cells. GPC 3 is highly expressed in HCC, which makes it an ideal target for HCC treatment. GPC3-CAR-T cells can effectively kill GPC3^+^ liver cancer cells, and their anti-tumor effect is proportional to the expression level of GPC3 (149). Batra et al. (146) designed GPC3-CAR-T cells that could co-express IL-15 and IL-21 to treat HCC. These CAR-T cells had superior expansion and persistence *in vitro* and *in vivo*, and the strongest anti-tumor activity *in vivo*. However, some obstacles limit the efficacy of CAR-T cells in the treatment of HCC. These obstacles include the lack of specific targets, homing barriers of CAR-T cells, inhibition of (TME), inhibition of immune checkpoints, etc. With the breakthrough of these obstacles, HCC patients will certainly get more benefits from CAR-T cell therapy.

**Table 3:** Some liver cancer-related CAR-T cell therapy targets.

Associated Malignancy	Target Antigens	Co-stimulating Domain	Generation of CAR-T	Author	Reference
	CD133	4-1BB	2^nd^	Wang et al.	(138)
	NKG2DL	4-1BB	2^nd^	Sun et al.	(139)
	GPC3	CD28	2^nd^	Wu et al.; Guo et al.	(140, 141)
		CD28,4-1BB	3^rd^	Jiang et al.	(142)
		CD28, ICOSL	3^rd^	Hu et al.	(143)
HCC		CD28, ICOSL,4-1BB	3^rd^	Hu et al.	(143)
		4-1BB	4^th^(CXCR2)	Liu et al.	(144)
		CD28	4^th^ (IL12)	Liu et al.	(145)
		CD28,4-1BB	4^th^ (IL15,IL21)	Batra et al.	(146)
	CD147	4-1BB	2^nd^	Zhang et al.	(147)
	AFP	CD28	2^nd^	Liu et al.	(148)

## Prospect and Summary

There remain various therapeutic methods for HCC, among which immunotherapy is playing an increasingly important role. The immunotherapy of HCC is in a rapid development stage. ICIs, tumor vaccines and ACT have great prospects and potential in the treatment of HCC. Combining different immunotherapy or immunotherapy with conventional treatment methods may produce synergistic effects (11). Nevertheless, the current clinical application of immunotherapy is relatively single, and its curative effect is limited. Consequently, it is necessary to strengthen the research of combined treatment mode. Immunotherapy, as an extremely promising therapy, brings new dawn to HCC patients. In the future, immunotherapy may become one of the mainstream methods of HCC.

## Author Contributions

LM: Writing- Original draft preparation, Investigation, table and figure preparation. ZZ: Investigation and table preparation. ZR: Investigation. YL: Conceptualization, Methodology, Supervision. All authors contributed to the article and approved the submitted version.

## Funding

This work was supported by Special Research Project of Lanzhou University Serving the Economic Social Development of Gansu Province (054000282) Lanzhou Talent Innovation and Entrepreneurship Project (2020-RC-38), the Fundamental Research Funds for the Central Universities (lzujbky-2020-kb14), and Major Science and Technology Special Project of Gansu Province (20ZD7FA003).

## Conflict of Interest

The authors declare that the research was conducted in the absence of any commercial or financial relationships that could be construed as a potential conflict of interest.

## Publisher’s Note

All claims expressed in this article are solely those of the authors and do not necessarily represent those of their affiliated organizations, or those of the publisher, the editors and the reviewers. Any product that may be evaluated in this article, or claim that may be made by its manufacturer, is not guaranteed or endorsed by the publisher.
